# Validation of the RayStation Monte Carlo dose calculation algorithm using realistic animal tissue phantoms

**DOI:** 10.1002/acm2.12733

**Published:** 2019-09-21

**Authors:** Andries N. Schreuder, Daniel S. Bridges, Lauren Rigsby, Marc Blakey, Martin Janson, Samantha G. Hedrick, John B. Wilkinson

**Affiliations:** ^1^ Provision Center for Proton Therapy – Knoxville Knoxville TN USA; ^2^ PhDRaySearch Laboratories Stockholm Sweden

**Keywords:** analytical dose algorithms, Monte Carlo, pencil beam scanning, proton therapy, spot scanning

## Abstract

**Purpose:**

The aim of this study is to validate the RayStation Monte Carlo (MC) dose algorithm using animal tissue neck phantoms and a water breast phantom.

**Methods:**

Three anthropomorphic phantoms were used in a clinical setting to test the RayStation MC dose algorithm. We used two real animal necks that were cut to a workable shape while frozen and then thawed before being CT scanned. Secondly, we made a patient breast phantom using a breast prosthesis filled with water and placed on a flat surface. Dose distributions in the animal and breast phantoms were measured using the MatriXX PT device.

**Results:**

The measured doses to the neck and breast phantoms compared exceptionally well with doses calculated by the analytical pencil beam (APB) and MC algorithms. The comparisons between APB and MC dose calculations and MatriXX PT measurements yielded an average depth difference for best gamma agreement of <1 mm for the neck phantoms. For the breast phantom better average gamma pass rates between measured and calculated dose distributions were observed for the MC than for the APB algorithms.

**Conclusions:**

The MC dose calculations are more accurate than the APB calculations for the static phantoms conditions we evaluated, especially in areas where significant inhomogeneous interfaces are traversed by the beam.

## 1 INTRODUCTION

The growth in proton therapy facilities worldwide has accelerated greatly in recent years,^1^, ^2^ mainly due to the clinical realization of pencil beam scanning (PBS) which increased the number of applicable treatments.^3^ Before the clinical utilization of PBS, proton therapy was limited to small and contiguous targets because the beam had to be shaped by an aperture and distally conformed to the target using a compensator. The increasing adoption of proton therapy is also a result of reducing the costs and footprint of a typical facility. Recently, Bortfeld and Loeffler^4^ argued that changes in current health care policies would further drive this clinical implementation. To accommodate this expansion, treatment planning systems had to improve and handle much more complicated anatomical sites. Our institution chose the RayStation treatment planning system (RaySearch Laboratories AB, Stockholm, Sweden) to handle this demand and was the first proton therapy facility to use RayStation for proton therapy treatments.^5^, ^6^

The first dose calculation models used for proton treatment planning employed raytracing methods which only considered the deposition of dose along straight lines traced from the source to the point of interest in the patient’s body. The deficiencies of these algorithms were well understood, but they were the only option in the early days when computers were still very slow. These algorithms were replaced by analytical pencil beam (APB) dose calculation algorithms as described by Petti^7^ and Hong.^8^ The first releases of the RayStation treatment planning system employed an APB algorithm for PBS that was partly based on the algorithm described by Soukup and Fippel.^9^ This APB dose engine divides the beam into many closely spaced mini‐beams, called “pencil beams”.^10^ Each pencil beam is calculated by factorization of the lateral proton fluence and the integrated depth dose along the central axis of the pencil beam. This factorization is only accurate given the infinite slab approximation in which the patient model is composed of semi‐infinite layers transverse to the central axis of the pencil beam. This assumption is not satisfied in the presence of lateral heterogeneity, causing erroneous dose calculation especially in lung targets. For the dose computation, the lateral proton fluence for each pencil beam is affected by stopping power, Multiple Coulomb Scattering, and nonelastic nuclear scattering. Summing all the individual pencil beams results in the total dose distribution.^11^

RaySearch Laboratories AB released a Monte Carlo (MC) dose calculation engine in May 2017 (US release) to supplement and eventually replace the APB algorithm currently used in the RayStation proton beam treatment planning system. In addition to improving the general accuracies of the proton beam dose calculations within the patient, the three other major improvements that the MC engine attempts are: (1) accurate dose calculation for targets in lung and inhomogeneous mediums, (2) accurate dose calculation when an aperture is used to sharpen the beam edge, and (3) accurate shallow dose spots in the presence of a range shifter when large patient‐to‐range shifter air gaps must be used. The latter two issues are straightforward to address during validation using standard water phantom measurements, but validating the dose in tissue, that is, a realistic clinical situation, is much harder.

This MC dose engine can be used for dose calculations for PBS and scattering‐based treatment deliveries. PBS deliveries allows for inverse treatment planning techniques where the weights of a large number of candidate spots are determined using single‐field optimization (SFO) or multifield optimization (MFO) strategies.^12^ In RayStation, the Monte Carlo dose engine is not only used for final dose computation of a given spot distribution but may also be used in the optimization of the plan. This means that the MC algorithm can be used to either calculate the final dose from APB‐optimized spot distributions, or it can be used for optimization and final dose calculation. The latter is the ideal option, but it can sometimes be time consuming especially during the initial phases of the planning process. RaySearch Laboratories AB explains:^11^

The MC engine can account for range shifters and apertures with arbitrary air gaps. A Class II transport algorithm is used for primary and secondary protons, while heavier secondary particles such as deuterons and alphas are transported only by taking energy loss into account using the Continuous Slowing Down Approximation (CSDA). [‘Class II’ methods classify interactions into “hard” and “soft” categories depending on energy: Interactions causing energy loss above a specified threshold (‘hard’ interactions) have their delta rays explicitly modeled, whereas less‐energetic interactions are summarized by sampling their condensed history (a statistical summary of multiple interactions).^13^] Neutral products such as neutrons and Gammas are not transported, but their fractions of absorbed energy are subtracted from the remaining beam. Delta electrons are considered to be stopped within 1 mm and are hence excluded from the beam’s calculation. The voxel grids of the simulation geometry are “characterized by [each voxel having] its own mass density, elemental composition, and mean ionization energy.” “Primary protons [are generated] in a plane upstream of the patient transport grid [or] most upstream beam modifier,” and they are transported “until they stop or [leave] the geometry. […] For increased performance, the integration of the ionization energy loss is carried out by a parameterized model of the Bethe‐Bloc[h] equation. […] Energy loss straggling is [accounted for with] the Bohr approximation [and] multiple scattering [uses] the theory of Goudsmit‐Saunderson. Nonelastic reactions are included by use of a data library of prestored tables of the needed quantities. The data library is compiled from data provided in the ICRU Report 63^14^ for a sequence of elements in the periodic table. The following quantities per element are used:

- total nonelastic cross section
- production cross section for proton, deuterons, and alpha particles
- fraction of incident energy leading to protons, deuterons, alphas, neutrals (neutrons and gammas), and heavy recoils (A> 4)
- double differential emission spectra for production of protons, deuterons, and alpha particles

When a new treatment modality such as PBS gains clinical utility, the question of treatment planning accuracy must be addressed. The physics of therapeutic proton beam dose calculations is generally less complicated than for x rays and electrons since most of the energy deposition mechanisms can be modeled by locally depositing the dose, at least within the voxel geometry typically used for clinical dose calculations. This allows for MC dose calculation engines to be simplified extensively, bringing down the computation times on standard computers to make treatment planning feasible using less expensive computers. Many such models have been developed over the years,^15^, ^16^ but it was only recently that RayStation was equipped with a clinical MC option.^17^ Recent reports by Saini et al. describe the process of commissioning the RayStation planning system for clinical PBS treatments^18^ and the dosimetric evaluation of the RayStation MC algorithm^19^ against phantom measurements and against the GATE^20^ MC dose engine. Recently Taylor et al.^21^ reported on the dose calculation inaccuracies revealed by measurements using the IROC (Imaging and Radiation Oncology Core, Houston, TX, USA) lung phantom. They found that when lung doses are calculated using APB, the target dose was overestimated by 7–46%. Earlier this year, Widesott et al. validated this MC algorithm for head‐and‐neck phantoms at multiple angles and air gaps at a single depth with single gamma analysis criteria.^22^ The need to validate and transition to RayStation’s MC dose algorithm is therefore clear.

Here we report on the more sophisticated clinical commissioning of the RayStation MC engine employing clinically realistic scenarios and accurate dose measurements in various anthropomorphic phantoms at multiple depths. We embarked on a series of experiments to validate the MC doses vs doses measured in the near‐reality phantoms for different geometries. Using animal tissues to validate dose calculations is a common method and yielded great results as described by Zheng,^23^ Grassberger,^24^ and Gurjar,^25^ though most of this work was done for passively scattered or uniformly scanned proton beams. Our aim was to develop phantoms that can validate the calculated dose “inside” the phantom and not “on the other side,” that is, a transmission‐type measurement. In addition to this study, we also verified the accuracy of MC calculations for lung; these findings are being prepared for subsequent publication.

We note that similar work has recently been published regarding the open‐source fast MC proton dose algorithm MCsquare.^26^, ^27^ In a recent experimental study involving a measured lateral profile measured in a water tank hosting a large lateral inhomogeneity, MCsquare was compared to an early research version of the RaySearch MC algorithm. It was found that both algorithms fared well in the study, but that RaySearch’s MC had a 2.5% better passing rate for a 2%/2 mm gamma criterion.^28^ With this experiment, we sought to build on this confidence of the reliability of this dose algorithm. Specifically, for the safety and improvement of our clinical practice we sought to answer the questions, “Does the current RayStation MC algorithm accurately predict the dose to breast and head‐and‐neck sites?” and, “What is the magnitude of improvement that MC provides over APB for dose distribution and range accuracy in breast and head‐and‐neck sites?”

## 2 METHODS

### 2.1 Dose validation phantoms

**Figure 1:**
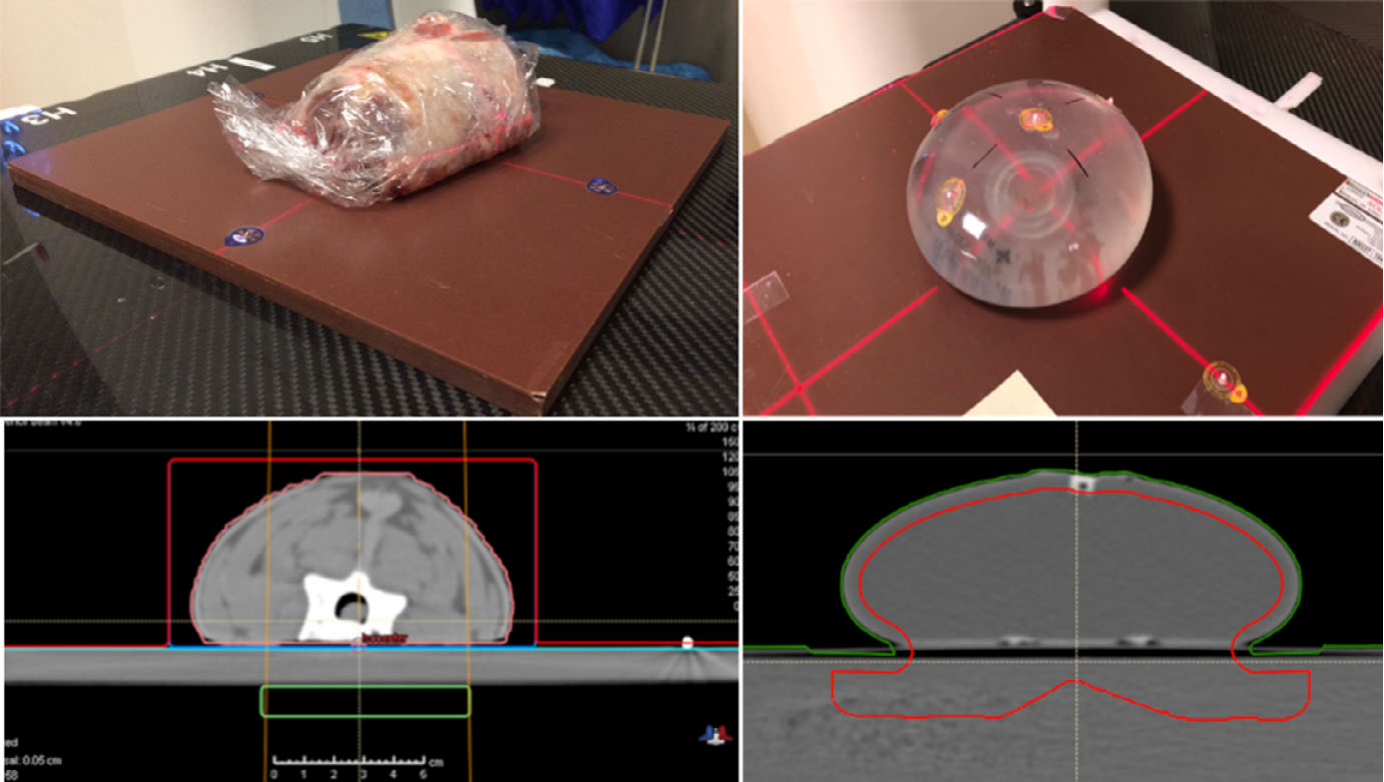
Representative phantoms used in this study: Lamb neck (left) and the water‐filled Mentor M + 350 cc sample prothesis (right) as seen by photography (upper) and computed tomography (lower). The green box in the left bottom pane and the red line in the lower‐right pane demarcates the dose optimization targets.

Beams were delivered using the IBA Universal Nozzle in a gantry treatment room at a proton center. The beams were delivered to the phantoms in a clinical setting, setup in the same manner that a patient is positioned for treatment. The lamb neck and breast phantoms are shown in Fig. 1. The deer neck was similar in appearance and treated in the same manner.

#### 2.1.1 Head and Neck (H&N) phantom

A lamb neck (Fig. 1) and a deer neck were used for the head‐and‐neck (H&N) phantoms. The necks were cut while frozen with a radial saw to have a flat surface just past the neck vertebrae. Both necks were then thawed and placed with their flat surfaces on 2‐mm water‐equivalent thick solid water slabs, which in turn were placed on the MatriXX PT (Ion Beam Applications S.A., Louvain‐la‐Neuve, Belgium) detector to measure the dose distal but in close proximity to the neck vertebrae. More solid water slabs were inserted between the phantom and the MatriXX PT to make measurements at deeper depths beyond the neck solid water interface for each phantom.

#### 2.1.2 Breast

The breast phantom (Fig. 1) was a water‐filled Mentor M + 350 cc sample prothesis (Mentor Worldwide LLC)^29^ placed on solid water slabs of different thicknesses.

### 2.2 Treatment planning

#### 2.2.1 CT SIM

The dose validation phantoms were scanned as real patients with computed tomography (CT) using a Siemens Somatom Definition AS CT scanner (Siemens Medical Solutions USA, Inc., Malvern, PA, USA). Care was taken to mark the phantoms for precise treatment planning and positioning in the proton beam. The dose grid resolution was set to 1 mm for all final dose computations, and the MC dose was computed to reach an average statistical uncertainty better than 0.5% for voxels with a dose higher than 50% of the maximum dose. The dose computation times required to calculate the final dose for each beam in this study using the MC and APB algorithms are listed in Table 1. The computations were done on a computer equipped with an Intel® Xenon CPU E‐5 v3 with a 2.3GHz dual processor. All the beams were optimized using the APB algorithm only since the purpose of the study was to compare the same beams, that is, identical spot distributions and spot doses. Re‐optimizing the beams with the MC algorithm would have resulted in slightly different spot distributions and spot doses due to the subtle differences in the algorithms addressed in this work.

**Table 1:** Dose calculation times compared for the MC and APB dose algorithms using uniform dose calculation grids of 1 mm and 2 mm, that is, 1 mm^3^ and 8 mm^3^ voxels.

Plan	Grid Size (mm)	Field	Dose calculation time (Sec)		Ratio (*t*_MC_/*t*_APB_)
			*t*_APB_	*t*_MC_	
Deer Neck	1	F1	20	209	10.5
	2	F1	7.3	35	4.8
	1	F2	16.6	168	10.1
	2	F2	7.4	30.8	4.2
Lamb Neck	1	F3	20.3	253	12.5
	2	F3	5.7	42.3	7.4
	1	F4	17.9	219	12.2
	2	F4	5.9	38.6	6.5

APB, analytical pencil beam.

#### 2.2.2 Dosimetry

**Figure 2:**
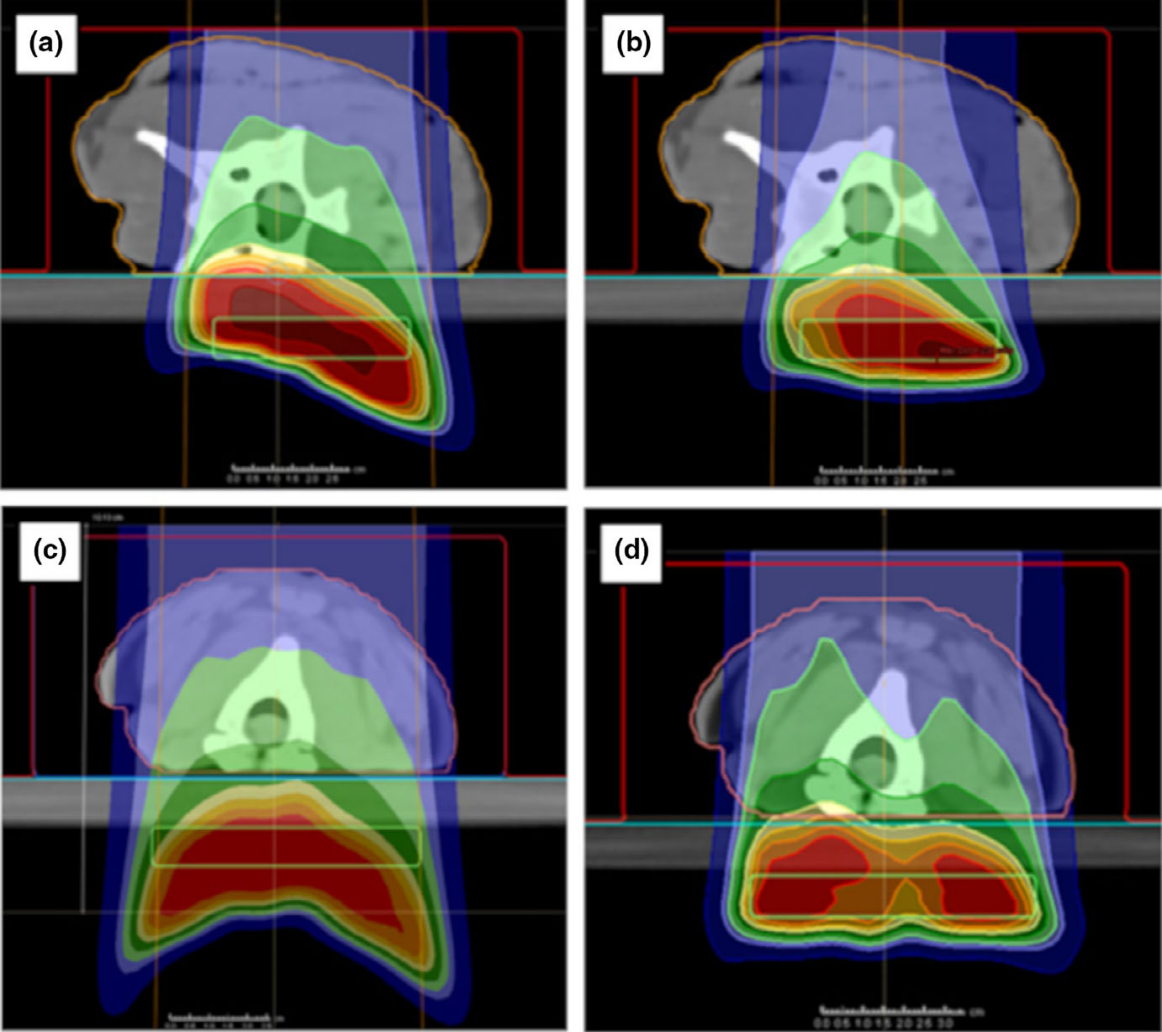
Dose distributions delivered to the realistic neck phantoms. Deer neck plans F1 and F2 are shown in panels A and B. The lamb neck plans, F3 and F4, are shown in panels C and D. The green boxes indicate the target regions used for the initial uniform dose plans that were modified as described in the text to obtain nonuniform dose distributions.

For the neck phantoms, the dose optimization targets were 1 × 5 × 7 cm^3^ volumes drawn in the solid water slabs at a depth posterior to the phantom solid water interface (the green boxes shown in Fig. 2). Two different AP beams, F1 and F3 for the lamb and deer neck phantoms respectively, were planned to deliver a deliberately nonuniform dose distribution beyond the animal tissue as shown in Fig. 2. We created plans using the APB algorithm for plan optimization.^30^, ^31^ The nonuniform dose distributions were accomplished by first overriding the material in the red volumes shown in Fig. 2, panels A and B, to water and optimizing the beam to deliver a uniform dose in the target (green boxes in Fig. 2). Secondly, the material override was then removed to recalculate the dose distributions resulting in a nonuniform distribution due to the presence of the heterogeneities in the neck phantoms. Two additional plans, F2 and F4, were created by copying plans F1 and F3 and removing random spots and layers from plans F1 and F3, respectively (Fig. 2 panels B and D). We selected these highly nonuniform plans because dose calculation accuracy is better tested by substantial gradients in the dose distribution. Particle therapy is best utilized when high gradients can be achieved to spare healthy tissue and deliver definitive dose next to an OAR. Hence dose gradients are necessary to consider for validating a dose calculation algorithm.

**Figure 3:**
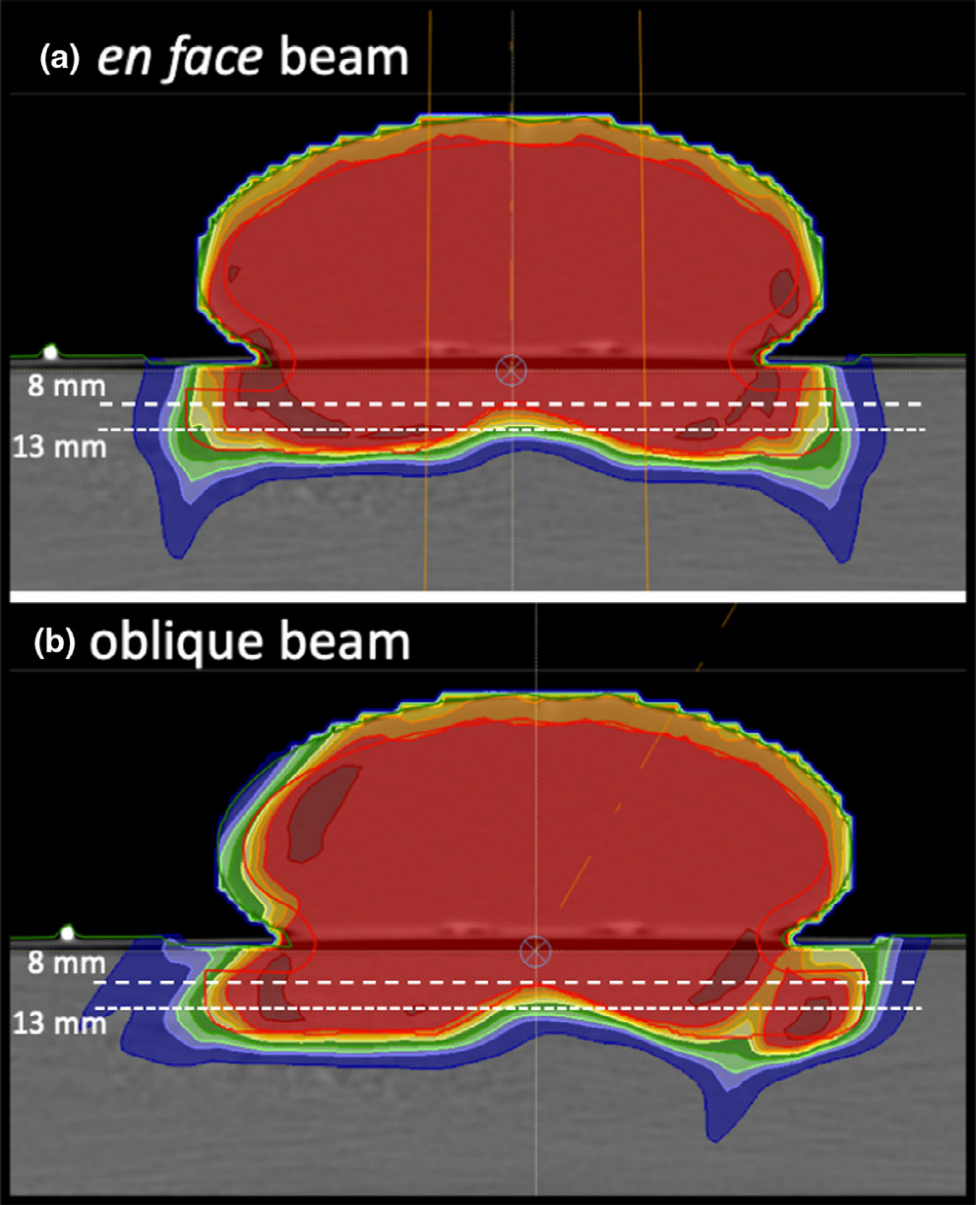
The breast phantom showing the beam orientations and measurement depths used. The red line demarcates the dose optimization target. The dose distributions shown were calculated with the APB algorithm. APB, analytical pencil beam.

The treatment plan for the breast phantom, shown in Fig. 3, followed a similar approach to the neck phantoms except that we drew an irregular target volume crossing over into the solid water to enable calculating and measuring dose beyond the breast prosthesis. We were primarily interested in the dose adjacent to the patient’s breast tissue, that is, the rib dose and at beam edges where the breast tissue forms a significant roll or other discrete soft tissue to air interface or oblique interface to the beam. Dose was optimized for the red target volume shown in Fig. 1 (bottom right panel) and Fig. 3 considering *en face* and oblique beams.

### 2.3 Measurements

#### 2.3.1 Phantom dose distributions

The dose distributions beyond the neck tissue were measured with the MatriXX PT detector at different depths beyond the interface of tissue and solid water while the beam was delivered to the phantom with a 2‐cm air gap between the phantom and a 7.5‐cm thick range shifter. The MatriXX PT detector has 6 mm of plastic proximal to the plane of measurement, causing the minimum water‐equivalent depth of measurement to be 6 mm. Accounting for this fact, our measurement depths were achieved by placing the MatriXX PT detector behind combinations of water‐equivalent solid water slabs. The measurement depths (*d_m_)* and solid water slabs used are tabulated in Table 2.

**Table 2:** Measurement depths for the phantom treatment plans.

Phantom	Plan name	Plan description	Measurement Depths, mm
Deer Neck	F1	First heterogeneous dose distribution	8, 26
Deer Neck	F2	Removing spots randomly from F1	8, 26
Lamb Neck	F3	Second heterogeneous dose distribution	8, 35
Lamb Neck	F4	Removing layers randomly from F3	8, 26
Breast		*en face*; oblique	8, 13

The water‐equivalent thickness ratio of the solid water material was 1.03. The phantom was aligned with the beam using the VeriSuite IGRT system (MedCom, Darmstadt, Germany) employing orthogonal X rays, exactly as is done for patients.

The MatriXX PT detector is used daily for patient specific QA measurements and is cross calibrated regularly in the reference TRS398 calibration beam.^33^ The TRS398 reference beam is calculated to deliver a uniform dose of 2 ± 1% Gy(RBE) in a 10 × 10 × 10 cm^3^ volume, that is, in the middle of a 10‐cm spread out Bragg peak (SOBP) with a range (distal 90% dose point) of 25 cm. The MatriXX PT is placed at a depth of 20 cm in a water phantom to measure the dose in the center of the calibration volume. The MatriXX PT calibration factor is set to report a dose of 2 Gy (RBE) at the measurement point while the field uniformity and symmetry is also verified. The flatfield calibration of MatriXX PT is also verified regularly in a large 6 MV X‐Ray field using an Elekta Synergy Linear accelerator.

### 2.4 Data analysis

Two‐dimensional (2D) and three‐dimensional (3D) gamma analyses of absolute doses were performed with the measured doses as reference, and the computed doses as comparison. Global gamma was considered where the 100% level was defined as the maximum dose of the computed doses.^34^ A gamma threshold of 5% and 10% was used for the 2D and 3D analyses respectively. This means that only measured doses above 5% (for the 2D analyses) and above 10% (for the 3D analyses) of the max dose were included in the analyses.

**Figure 4:**
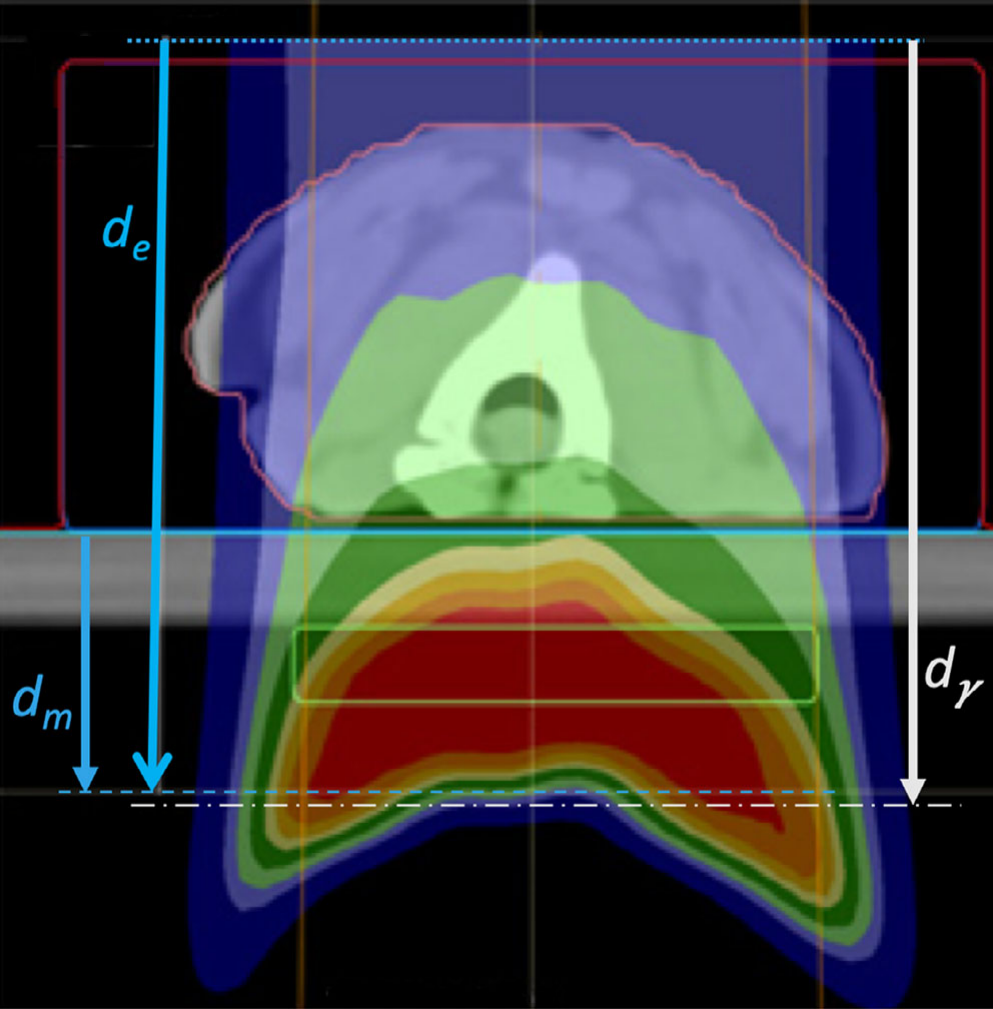
A schematic showing the "expected DICOM depth" *d_e_*, that is, the depth in the DICOM dose file at which we expect the best gamma index agreement given accurate dose calculation; the depth of measurement *d_m_*; and the depth of best gamma‐index agreement *d_γ_*. *d_e_* is measured from the anterior surface of the dose cube (dotted line) to the placement of the measuring plane inside the MatriXX PT (blue dashed line). *d_m_* is measured from the solid water surface (blue line) to the same position. *d_γ_* is determined by varying the dose calculation plane until best agreement is obtained with our in‐house software given the reference position of solid water surface (blue line).

The MatriXX PT measurements were exported from OmniPro I’mRT (Ion Beam Applications S.A., Louvain‐la‐Neuve, Belgium) as Omni Pro Generic (OPG) ASCII files. All the dose files were imported into our in‐house gamma analysis software which compares a computed 2D dose plane extracted from a dose cube at a certain depth to the corresponding measured dose planes obtained from various 2D dose measuring devices. In this 2D gamma analysis, the MatriXX PT’s 7‐mm‐resolution measurements were linearly interpolated to a 2‐mm grid. Because the MatriXX PT device has been cross calibrated to yield absolute dose, the 2D gamma analysis was performed without prior normalization of the measured or calculated doses. The measured 2D dose planes for the beams delivered to the phantoms were compared to the APB‐ and MC‐calculated doses with gamma parameters of 2%/2 mm, 3%/3 mm, and 5%/3 mm using global gamma analyses. We started the 2D comparisons by extracting the first dose plane from the dose cube at the expected depth (*d_e_*), which is the depth in the calculated cube where the measurement was done. The *d_e_* was obtained by locating the measured 2D dose plane (*d_m_*) in the planning system and then measuring the distance from *d_m_* to the edge of the dose calculation grid in the direction of the beam. The *d_e_* is therefore the depth in the DICOM dose cube produced by the planning system. For each 2D comparison, the depth of best gamma agreement (*d_γ_*) was found by iteratively modifying the depth of the dose plane extracted from the calculated dose cube until the best agreement was found with *d_m_*. These depths are illustrated in Fig. 4. We follow this same method for patient specific QA validations for real patient treatments.

We also performed 3D gamma analyses of all the MatriXX PT measured data using tools in the RaySearch Laboratories dose engine validation test suite. The 3D gamma analyses were performed between the measured dose plane and the calculated dose plane extracted at the expected depth (*d_e_*). The gamma analysis tools in this internal software package are the same as those included in the clinically available product Compass from IBA Dosimetry and RaySearch.^32^ In this gamma analysis, the sparsely measured data points were used as the reference dose with the computed 3D dose as evaluation, which is the converse of the 2D gamma analyses described above.

## 3 RESULTS

### 3.1 Dose calculation times

The dose calculation times required by the MC algorithm (*t*_MC_) are significantly longer than for APB algorithm (*t*_APB_) and scales approximately as the inverse cube of the dose computation grid size. The ratio of *t*_MC_/*t*_APB_ is also listed in Table 1 showing that the MC calculation times are on average 5.7 (±1.5 SD) and 11.3 (± 1.2 SD) times longer for beams using 2‐ and 1‐mm grid spacings, respectively. It is expected that *t*_MC_ will be reduced significantly when GPU‐based calculations become available in future releases of the RayStation software.

### 3.2 Gamma analyses

The gamma passing rates for the animal neck phantoms and breast phantom are listed in Tables 3 and 4. The depths of best 2D gamma agreement for the neck phantoms are also listed in Table 3. We report the 3D gamma pass rates at the expected measurement planes.

**Table 3:** The 2D and 3D Gamma analysis results for the animal tissue neck phantoms.

	Plan & Beam	Plane Depth, *d_m_* (mm)	Expected DICOM Depth, *d_e_* (mm)	Best Gamma depth, *d_γ_* (mm)	*d_e_* ‐ *d_γ_* (mm)	In‐House 2D Analyses			RaySearch Lab.		
						gamma agreement, %			gamma agreement, %		
						2D: 2%/2 mm	2D: 3%/3 mm	2D: 5%/3 mm	3D: 2%/2 mm	3D: 3%/3 mm	3D: 5%/3 mm
Analytical Pencil Beam Algorithm (APB)	Deer F1	8.0	71.0	71.0	0.0	90.2	97.9	99.8	96.9	99.2	99.2
		26.0	89.0	88.0	1.0	78.1	91.4	95.1	95.1	99.2	99.2
	Deer F2	8.0	71.0	71.0	0.0	91.4	97.6	99.6	99.2	99.2	100.0
		26.0	89.0	88.3	0.7	64.7	75.7	78.3	98.5	100.0	100.0
	Lamb F3	8.0	74.1	74.1	0.0	92.6	99.5	100.0	98.1	100.0	100.0
		35.0	101.3	101.8	−0.5	76.8	91.7	96.5	100.0	100.0	100.0
	Lamb F4	8.0	74.1	74.1	0.0	91.9	99.4	99.9	98.9	99.4	99.4
		26.0	91.3	91.3	0.0	59.5	75.7	78.2	82.5	97.7	97.7
					Avg.	80.7	91.1	93.4	96.1	99.4	99.5
Monte Carlo Algorithm (MC)	Deer F1	8.0	71.0	73.0	−2.0	82.6	96.8	98.7	100.0	100.0	100.0
		26.0	89.0	88.3	0.7	65.8	91.0	95.6	100.0	100.0	100.0
	Deer F2	8.0	71.0	71.4	−0.4	93.8	99.4	99.9	100.0	100.0	100.0
		26.0	89.0	89.4	−0.4	74.2	88.9	91.5	100.0	100.0	100.0
	Lamb F3	8.0	74.1	74.1	0.0	96.2	99.9	100.0	98.7	100.0	100.0
		35.0	101.3	101.3	0.0	76.7	95.5	97.6	100.0	100.0	100.0
	Lamb F4	8.0	74.1	74.1	0.0	98.4	100.0	100.0	100.0	100.0	100.0
		26.0	91.3	91.8	−0.5	68.4	87.8	93.0	93.8	99.4	100.0
					Avg.	81.9	94.9	97.0	99.1	99.9	100.0

The differences in the expected DICOM depth (*d_e_*_)_ and the depth of best gamma agreement (*d_γ_*) for the 2D analyses are also listed.

**Table 4:** 3D Gamma pass rates for the breast phantom.

	*d_m_* (mm)	*d_e_ = d_γ_* (mm)	Criteria	Pass Rate, %	
				MC	APB
*En face* beam	8	61.3	2 mm/2%	93.82	86.87
			3 mm/3%	98.84	93.44
			3 mm/5%	99.23	94.21
	13	66.3	2 mm/2%	**94.94**	89.49
			3 mm/3%	**99.61**	94.94
			3 mm/5%	99.61	96.11
Oblique beam	8	61.3	2 mm/2%	94.41	86.51
			3 mm/3%	97.7	92.76
			3 mm/5%	**99.67**	94.74
	13	66.3	2 mm/2%	89.76	87.03
			3 mm/3%	98.29	95.22
			3 mm/5%	99.66	97.95

APB, analytical pencil beam.

The depth of best gamma agreement *d_γ_* was found to be the expected DICOM depth *d_e_* in the dose cube; the corresponding depth relative to the solid water surface *d_m_* is also tabulated. Bold pass rate percentages are the best agreement per criteria.

### 3.3 Neck phantoms

**Figure 5:**
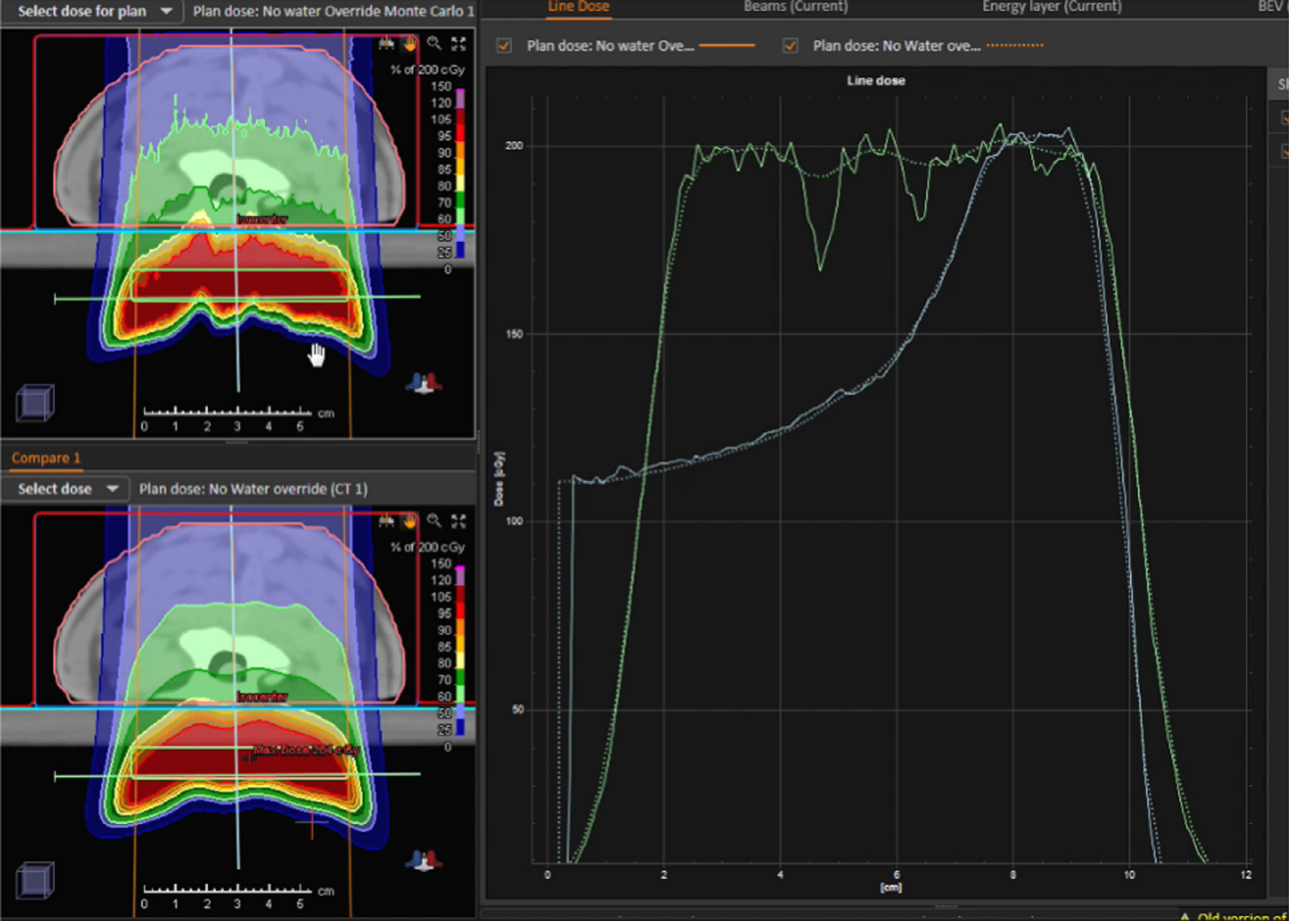
A comparison of dose distributions calculated by the Monte Carlo algorithm (upper left) and analytical pencil beam algorithm (lower left) for the lamb neck phantom in the region where the largest differences were observed. The depth dose and lateral dose profiles along the vertical pale blue and horizontal green lines in the left panels are shown in the right pane for the MC (solid) and APB (dotted) doses. APB, analytical pencil beam.

Calculated dose distributions for the F3 lamb neck plan are shown in Fig. 5. for the axial slice where the largest disagreements were observed. MC‐calculated dose was about 20% less than APB‐calculated dose in some areas due to the bone tissue interfaces. The upper left panel shows the MC dose while the bottom left panel shows the APB dose. The line profiles shown in the right panel were extracted along the solid straight lines shown in the left panels. The APB algorithm has problems in the vicinity of extreme density variations caused by the air‐bone interface (Fig. 5). It is important to notice that the depth doses shown by the blue lines in the right panel of Fig. 5 are not dramatically affected by the heterogeneity.

**Figure 6:**
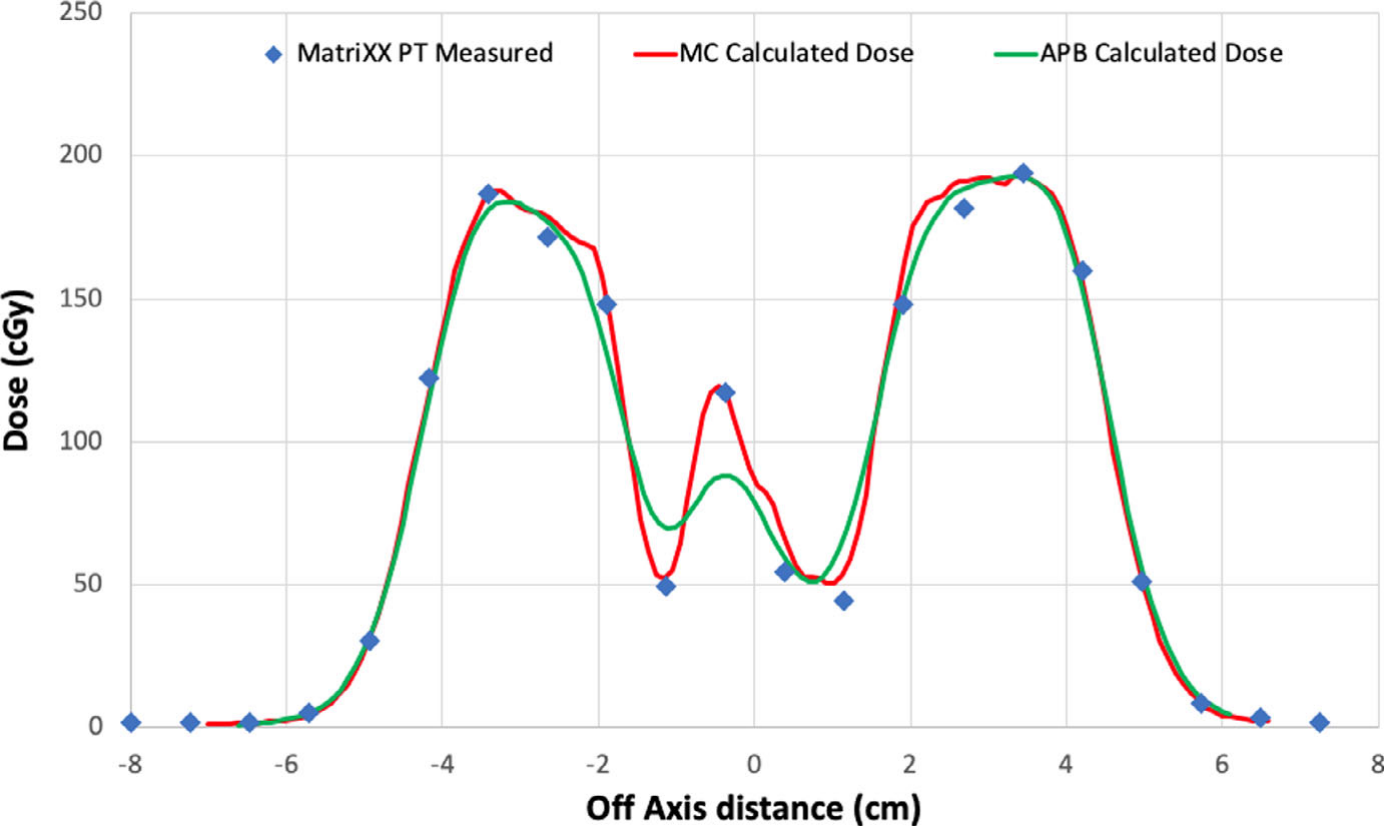
Lateral profiles comparing the MatriXX PT measured (blue triangles), MC calculated (red lines), and APB calculated (green lines) at a measurement depth of 35 mm in solid water (Dicom depth = 101.3 mm) for the lamb neck phantom plan F3. APB, analytical pencil beam.

**Figure 7:**
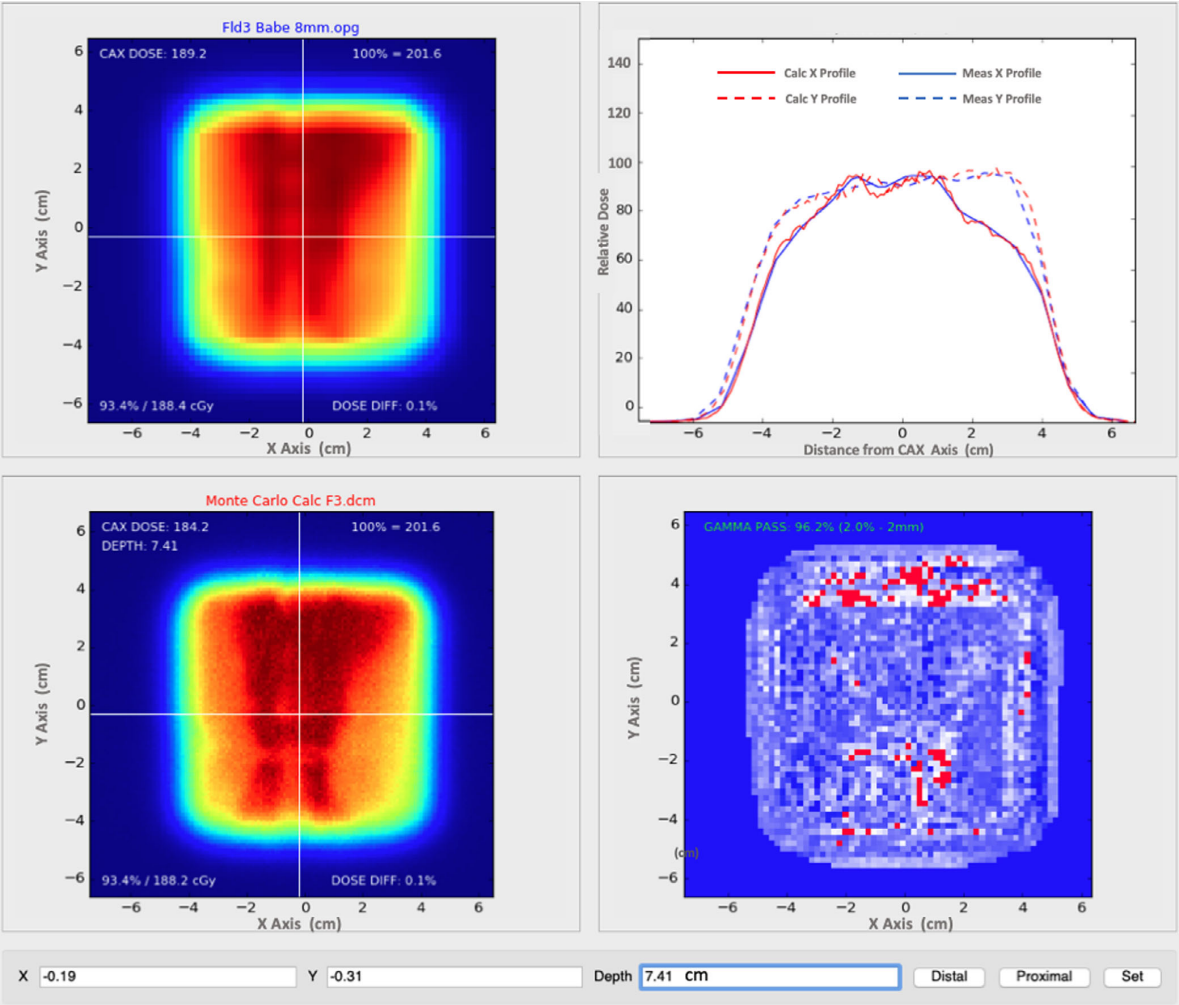
Our in‐house gamma analysis software comparing MC dose (lower left) at a depth of 8 mm beyond the lamb phantom via the MatriXX PT (upper left) with 2‐mm grid interpolation. Agreement at 2%/2 mm is 96.2% at a DICOM depth of 74.1 mm from the anterior edge of the dose cube (lower right). Dose profiles in the lateral (solid lines) and longitudinal (dashed lines) central axes are also displayed for the MC dose (red) and measured dose (blue) in the remaining pane (upper right). This depth is anterior to the Bragg peak.

**Figure 8:**
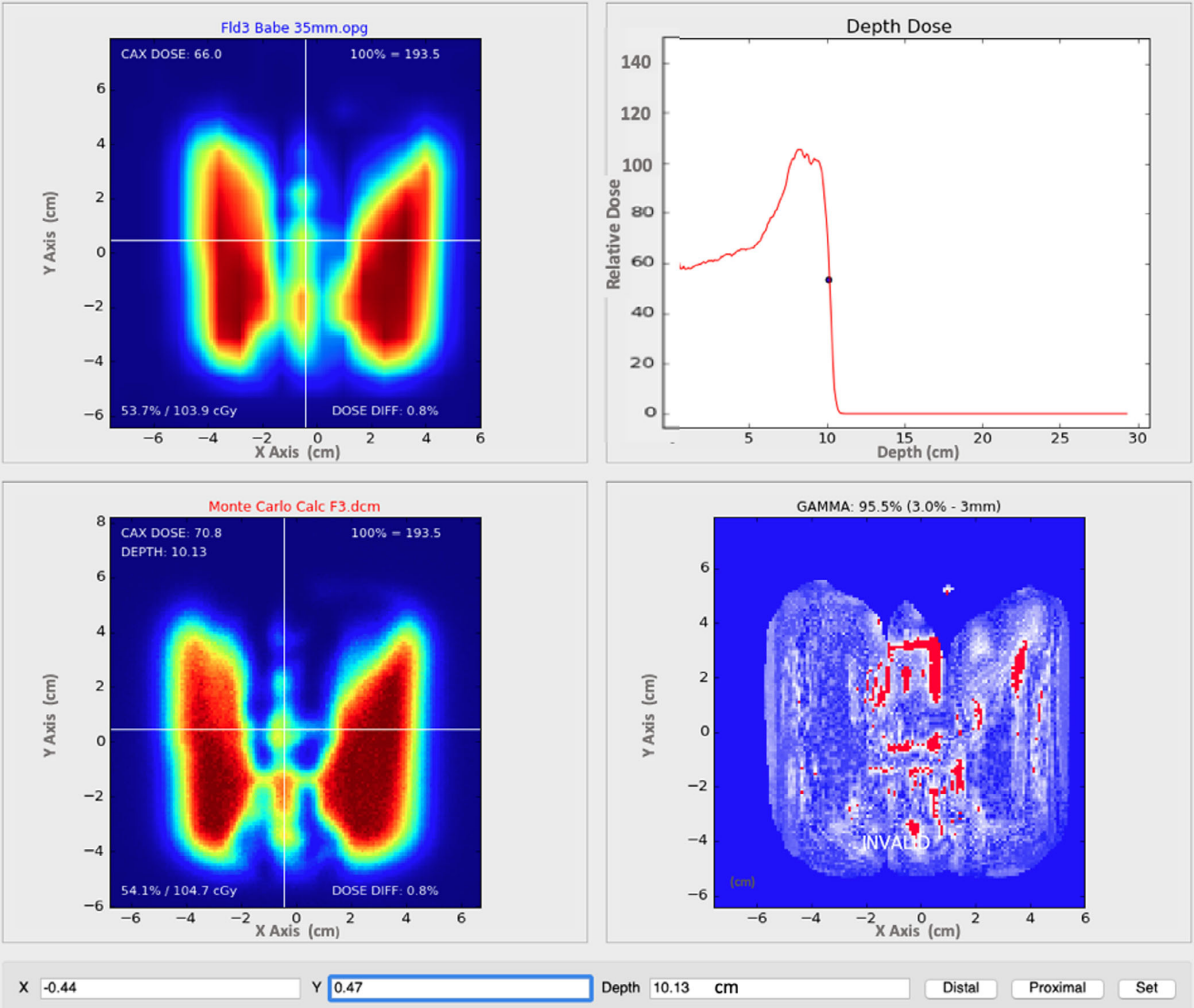
Our in‐house gamma analysis software comparing MC dose (lower left) at a depth of 35 mm beyond the lamb phantom via the MatriXX PT (upper left) with 2‐mm grid interpolation. Agreement at 3%/3 mm is 96.2% at a DICOM depth of 101.2 mm from the anterior edge of the dose cube (lower right). This depth is within the Bragg peak falloff (upper right).

Lateral dose profiles calculated with MC and APB for the F3 lamb neck plan at a depth of 35 mm in solid water corresponding to the expected DICOM depth of 101.3 mm are compared with the corresponding measured profile in Fig. 6. Screenshots from our in‐house 2D gamma analysis software are shown in Fig. 7 and Fig. 8 for the F3 plan of the lamb neck phantom at the two measurement depths of 8 mm (expected DICOM depth = 74.1 mm) and 35 mm (expected DICOM depth = 101.3 mm) in solid water. The bottom right panels in Fig. 7 and Fig. 8 show the gamma analyses panes. The upper right panel in Fig. 7 shows the X and Y profile comparisons along the lines indicated in the left panels at a depth of 7.41 cm. The red lines are the MC‐calculated doses while the blue lines are from the measured data. The upper right panel of Fig. 8 shows the depth‐dose profile extracted from the MC‐calculated dose cube at the point of the cursor in the left panels (shown with the white lines in the left panels). The blue dot shows the depth at which the analyses were done. As one can see, the analysis depth of 101.2 mm is in the distal edge of the beam.

### 3.4 Breast phantom

For the breast plans, only 3D gamma analysis was performed. The passing rates are listed in Table 4.

## 4 DISCUSSION

### 4.1 Gamma analysis

In each 2D gamma analysis, we looked for the depth in the calculated dose cube where the best gamma parameters were obtained, that is, the depth in the phantom where the measured and calculated dose distributions most agreed. We refer to that depth as the depth of best gamma agreement (*d_γ_*). The expected DICOM depth (*d_e_*) is the depth of the effective measurement plane within the dose cube as illustrated in Fig. 4. The differences between *d_γ_* and *d_e_* are included in Table 3 and are less than 1 mm in all but one case. This exceptional agreement between *d_γ_* and *d_e_* indicates the accuracy of the dose calculation from the perspective of range and dose distribution. The 2D gamma shows mixed results in this study. While the 2D:3%/3 mm results for the MC dose engine are generally better than the corresponding passing rates for the APB, the 2D:2%/2 mm passing rates are similar and inconclusive. This is primarily due to the fact that the two dose algorithms agree fairly well over most of the calculation volume except for regions of discrete tissue inhomogeneities as can be seen in Fig. 5 for the bone–air interface region of the lamb neck phantom. Since the phantoms were small, even significant dose disagreements in a small volume of the dose calculation volume might not affect the gamma score significantly. Evaluating the dose along discrete dose lines through the calculation volume is a better method to derive conclusions about the calculation accuracy. This is illustrated in Fig. 6, which shows the measured vs. calculated differences beyond the bone–air interface region of the lamb neck phantom.

The 3D gamma passing rates of the neck phantoms in Table 3 tells a different story. The average 3D:2%/2 mm passing rate for the APB dose engine is a remarkably high 96%, and the corresponding result for MC is 99%. The average passing rates for the less strict gamma criteria are >99% for both dose engines. For the breast cases (Table 4), the MC dose results are consistently better than the APB for all plans, depths and gamma criteria, with passing rates >93% for all but one analysis point (2%/2 mm pass rate = 89.8% for the oblique beam at 13 mm). However, the APB results are still good, with 3%/3 mm passing rates better than 92% for all cases.

This work reveals the importance of 3D gamma analyses. The 2D gamma analyses often give weaker results and might be interpreted as not acceptable. Since proton therapy is by nature a three‐dimensional problem, comparing doses in only two dimensions does not properly consider the statistical variation of dose with depth. Multiple 2D gamma analyses do have a 3D component in the sense that we sought for the best 2D comparisons in dose as a function of depth, that is, we selected the depths where the measured dose plane compared the best with the calculated plane. The problem is that this method is uni‐directional and does not evaluate the discrepancies in both directions, for example, shallower and deeper in the calculated cube. The 3D analyses also evaluated the distance to agreement (DTA) in the depth direction. In steep dose‐gradient areas, the DTA in the transverse plane might be large, leading to a weak gamma index, while the DTA in the depth direction can be smaller. The converse is also true. The gamma index might be in good agreement at the beam edge, as is often the case for proton beams, but poor agreement inside the field might exist mainly due to inhomogeneities.

### 4.2 Benefits of the Monte Carlo dose calculations

The data shown in this report validates the use of the MC dose calculation engine in RayStation for clinical use. The main benefits of a MC dose engine are for dose calculations in lung and for beams where a range shifter and larger air gap is required. We investigated these as well and the work will be presented in a subsequent study. In this study, the air gap was kept rather small and the lateral inhomogeneities of the phantoms were limited. Consequently, the accuracy of the APB dose algorithm was found to be acceptable (in terms of passing rates of 3%/3‐mm gamma analysis), although the MC dose accuracy was consistently better. The differences in the dose calculations from the APB and MC dose engines are very similar for the most part. Significant differences in dose (>=24%) occur after the beam traverses discrete density changes as seen in Fig. 5 and Fig. 6. It is important to note that the largest differences are observed for single beams in the head‐and‐neck region, however, single beams are not often used in the clinical setting. These kinds of interface errors are diminished by using multiple beams traversing the interface at different angles.

We note also the overall difference in dose conformality. MC calculations are more granular whereas the APB calculations appear smoother. This is because the APB algorithm is using the infinite slab approximation as described earlier, which is clearly not addressing the density interfaces correctly. This is most likely the primary reason why the MC calculations struggle to demonstrate superiority over APB calculations at the stricter gamma criteria and more so for the 2D analyses. In areas where there are no discrete density changes, the calculations agree very well.

## 5 CONCLUSION

In this work we validated the RayStation 6 Monte Carlo and APB dose calculation algorithms for head‐and‐neck and breast phantoms. The MC results were systematically better than the APB results when compared in a 3D fashion, although APB was found to be clinically acceptable for the studied cases. We further demonstrated depth‐dose discrepancy to be less than 1% for both algorithms. This work also highlighted the spatial limitation of 2D gamma, supporting the use of 3D gamma analyses for evaluating 3D dose distributions. Similar to isodose curves complementing dose‐volume histograms, we must pay attention to where the gamma‐index criteria are not satisfied in addition to the relative percentage to which it is satisfied.

We recommend implementing the RayStation Monte Carlo algorithm as a direct means to improve accuracy in treatment planning. Our future work will discuss the influence of air gap, range shifters, and apertures on this algorithm’s accuracy. We will also validate this algorithm for targets in lung tissue using a novel phantom allowing dose measurement within a realistic tumor phantom.

## CONFLICT OF INTEREST

The authors have no relevant conflict of interest to disclose.

## References

[1] Durante MORLJ . Charged‐particle therapy in cancer: clinical uses and future perspectives. Nat Rev: Clin Oncol. 2017;14:483–495. 28290489 10.1038/nrclinonc.2017.30

[2] Particle Therapy Co‐Operative Group . Particle therapy facilities in operation. Particle Therapy Co‐Operative Group. 2018. Available at: https://ptcog.ch/index.php/facilities‐in‐operation. Accessed November 29, 2018.

[3] Schreuder AN , Hedrick SG , Renegar JR , et al. A review of proton radiation therapy and the path to widespread clinical adoption. Med Phys Int J. 2016;4:35–46.

[4] Bortfeld TR , Loeffler JS . Three ways to make proton therapy affordable. Nature. 2017;549:451–453. 28959981 10.1038/549451a

[5] Löf J . First PBS/IMPT Proton Treatments with RayStation. 2014. Available at: https://www.raysearchlabs.com/media/press‐old/?year=2014&cisionid =1629464. Accessed November 26, 2018.

[6] Löf J . Provision CARES Proton Therapy Center in Knoxville, Tennessee, US, Treats First Patients Using RayStation and the New ProNova SC360 Proton System. July 3, 2018. Available at: https://www.raysearchlabs.com/press/?year=2018&cisionxml:id=2981630. Accessed November 26, 2018.

[7] Petti P . Differential‐pencil‐beam dose calculations for charged particles. Med Phys. 1992;19:137–149. 1320182 10.1118/1.596887

[8] Hong L , Michael G , Bucciolini M , et al. A pencil beam algorithm for proton dose calculations. Phys Med Biol. 1996;41:1305–1330. 8858722 10.1088/0031-9155/41/8/005

[9] Soukup M , Fippel M , Alber M . A pencil beam algorithm for intensity modulated proton therapy derived from Monte Carlo simulations. Phys Med Biol. 2005;50:5089–5104. 16237243 10.1088/0031-9155/50/21/010

[10] Stein J , Swift R , Inventor . Radiant energy imaging with scanning pencil beam. US Patent # US3,780,291A. Dec 18, 1973.

[11] RaySearch Laboratories AB . RayStation 6 reference manual. Stockholm: RaySearch Laboratories AB; 2017.

[12] Quan E , Liu W , Wu R , et al. Preliminary evaluation of multifield and single‐field optimization for the treatment planning of spot‐scanning proton therapy of head and neck cancer. Med Phys. 2013;40:081709. 23927306 10.1118/1.4813900 PMC3732307

[13] Parodi K . Monte Carlo methods for dose calculations In: Linz U, ed. Ion Beam Therapy: Fundamentals, Technology, Clinical Applications. New York, NY: Springer; 2012:97–116.

[14] International Commission on Radiation Units & Measurements, Inc . Nuclear Data for Neutron and Proton Radiotherapy and for Radiation Protection (Report 63). Available at: https://icru.org/home/reports/nuclear‐data‐for‐neutron‐and‐proton‐radiotherapy‐and‐for‐radiation‐protection‐report‐63. Accessed January 15, 2019.

[15] Fippel M , Soukup M . A Monte Carlo dose calculation algorithm for proton therapy. Med Phys. 2004;31:2263–2273. 15377093 10.1118/1.1769631

[16] Jia X , Schümann J , Paganetti H , Jiang S . GPU‐based fast Monte Carlo dose calculation for proton therapy. Phys Med Biol. 2012;57:7783–7797. 23128424 10.1088/0031-9155/57/23/7783 PMC4474737

[17] RaySearch Laboratories AB .Proton Monte Carlo dose calculation now part of RayStation. RaySearch Labs. May 10, 2017. Available at: https://www.raysearchlabs.com/media/press/2017/proton‐monte‐carlo‐dose‐calculation‐now‐part‐of‐raystation/. Accessed November 13, 2018.

[18] Saini J , Cao N , Bowen SR , et al. Clinical commissioning of a pencil beam scanning treatment planning system for proton therapy. Int J Part Ther. 2016;3:51–60. 10.14338/IJPT-16-0000.1 PMC6871575 31772975

[19] Saini J , Maes D , Egan A , et al. Dosimetric evaluation of a commercial proton spot scanning Monte‐Carlo dose algorithm: comparisons against measurements and simulations. Phys Med Biol. 2017;52:7659–7681. 10.1088/1361-6560/aa82a5 28749373

[20] Jan S , Santin G , Strul D , et al. GATE: a simulation toolkit for PET and SPECT. Phys Med Biol. 2004;49:4543–4561. 15552416 10.1088/0031-9155/49/19/007 PMC3267383

[21] Taylor PA , Kry SF , Followill DS . Pencil beam algorithms are unsuitable for proton dose calculations in lung. Int J Radiat Oncol Biol Phys. 2017;99:750–756. 28843371 10.1016/j.ijrobp.2017.06.003 PMC5729062

[22] Widesott L , Lorentini S , Fracchiolla F , Farace P , Schwarz M . OC‐0087: clinical validation of Monte Carlo dose calculation for pencil beam scanning proton therapy. Radiother Oncol. 2018;127:S45–S46.

[23] Zheng Y , Kang Y , Zeidan O , Schreuder N . An end‐to‐end assessment of range uncertainty in proton therapy using animal tissues. Phys Med Biol. 2016;61:8010–8024. 27779135 10.1088/0031-9155/61/22/8010

[24] Grassberger C , Daartz J , Dowdell S , Ruggieri T , Sharp G , Paganetti H . Quantification of proton dose calculation accuracy in the lung. Int J Radiat Oncol Biol Phys. 2014;89:424–430. 24726289 10.1016/j.ijrobp.2014.02.023 PMC4028367

[25] Gurjar OP , Mishra SP , Bhandari V , Pathak P , Patel P , Shrivastav G . Radiation dose verification using real tissue phantoms in modern radiotherapy techniques. J Med Phys. 2014;39:44–49. 24600172 10.4103/0971-6203.125504 PMC3931228

[26] Huang S , Souris K , Li S , et al. Validation and application of a fast Monte Carlo algorithm for assessing the clinical impact of approximations in analytical dose calculations for pencil beam scanning proton therapy. Med Phys. 2018;45:5631–5642. 30295950 10.1002/mp.13231

[27] Université catholique de Louvain . Fast Monte Carlo simulations for proton PBS. Available at: http://www.openmcsquare.org. Accessed November 26, 2018.

[28] Sorriaux J , Testa M , Paganetti H , et al. Experimental assessment of proton dose calculation accuracy in inhomogeneous media. Physica Med. 2017;38:10–15. 10.1016/j.ejmp.2017.04.020 28610689

[29] Mentor Worldwide LLC. about us . Available at: http://www.mentorwwllc.com/global‐us/AboutUs.aspx. Accessed November 27, 2018.

[30] Raysearch Laboratories AB . RayStation 6 SP1 Release Notes; 2017.

[31] Raysearch Laboratories AB . RAYSTATION 6 SP1: Instructions for Use; 2017.

[32] IBA .Compass: 2‐in‐1 Patient Dose QA in 3D Patient Anatomy. iba Dosimetry. Available at: https://www.iba‐dosimetry.com/product/compass/. Accessed November 16, 2018.

[33] Andreo P , Burns DT , Hohlfeld K , et al.; Iaea International Atomic Energy Agency . “Absorbed Dose Determination in External Beam Radiotherapy. An International Code of Practice for Dosimetry Based on Standards of Absorbed Dose to Water”, Technical Report Series no. 398. Vienna 2000.

[34] Low DA , Dempsey JF . Evaluation of the gamma dose distribution comparison method. Med Phys. 2003;30:2455–2464. 14528967 10.1118/1.1598711

